# Stop and Play Digital Health Education Intervention for Reducing Excessive Screen Time Among Preschoolers From Low Socioeconomic Families: Cluster Randomized Controlled Trial

**DOI:** 10.2196/40955

**Published:** 2023-05-04

**Authors:** Diana Raj, Norliza Ahmad, Nor Afiah Mohd Zulkefli, Poh Ying Lim

**Affiliations:** 1 Department of Community Health Faculty of Medicine and Health Sciences Universiti Putra Malaysia Serdang Malaysia; 2 Ministry of Health Malaysia Putrajaya Malaysia

**Keywords:** child, preschool, screen time, mother-child, randomized controlled trial, mobile phone

## Abstract

**Background:**

High prevalence of excessive screen time among preschool children is attributable to certain parental factors such as lack of knowledge, false perception about screen time, and inadequate skills. Lack of strategies to implement screen time guidelines, in addition to multiple commitments that may hinder parents from face-to-face interventions, demands the need to develop a technology-based parent-friendly screen time reduction intervention.

**Objective:**

This study aims to develop, implement, and evaluate the effectiveness of *Stop and Play*, a digital parental health education intervention to reduce excessive screen time among preschoolers from low socioeconomic families in Malaysia.

**Methods:**

A single-blind, 2-arm cluster randomized controlled trial was conducted among 360 mother-child dyads attending government preschools in the Petaling district, who were randomly allocated into the intervention and waitlist control groups between March 2021 and December 2021. This 4-week intervention, developed using whiteboard animation videos, infographics, and a problem-solving session, was delivered via WhatsApp (WhatsApp Inc). Primary outcome was the child’s screen time, whereas secondary outcomes included mother’s screen time knowledge, perception about the influence of screen time on the child’s well-being, self-efficacy to reduce the child’s screen time and increase physical activity, mother’s screen time, and presence of screen device in the child’s bedroom. Validated self-administered questionnaires were administered at baseline, immediately after the intervention, and 3 months after the intervention. The intervention’s effectiveness was evaluated using generalized linear mixed models.

**Results:**

A total of 352 dyads completed the study, giving an attrition rate of 2.2% (8/360). At 3 months after the intervention, the intervention group showed significantly reduced child’s screen time compared with the control group (β=−202.29, 95% CI −224.48 to −180.10; *P*<.001). Parental outcome scores also improved in the intervention group as compared with that in the control group. Mother’s knowledge significantly increased (β=6.88, 95% CI 6.11-7.65; *P*<.001), whereas perception about the influence of screen time on the child’s well-being reduced (β=−.86, 95% CI −0.98 to −0.73; *P*<.001). There was also an increase in the mother’s self-efficacy to reduce screen time (β=1.59, 95% CI 1.48-1.70; *P*<.001) and increase physical activity (β=.07, 95% CI 0.06-0.09; *P*<.001), along with reduction in mother’s screen time (β=−70.43, 95% CI −91.51 to −49.35; *P*<.001).

**Conclusions:**

The *Stop and Play* intervention was effective in reducing screen time among preschool children from low socioeconomic families, while improving the associated parental factors. Therefore, integration into primary health care and preschool education programs is recommended. Mediation analysis is suggested to investigate the extent to which secondary outcomes are attributable to the child’s screen time, and long follow-up could evaluate the sustainability of this digital intervention.

**Trial Registration:**

Thai Clinical Trial Registry (TCTR) TCTR20201010002; https://tinyurl.com/5frpma4b

## Introduction

### Background

Screen time is defined as the time occupied by screen-based activities on visual devices such as television, smartphones, computers, video games, tablets and iPads, and other handheld gadgets [1]. The World Health Organization recommends a limit of 1 hour of screen time per day for children aged 2 to 5 years [2]. Nevertheless, the prevalence of children exceeding the limit is increasing, making it an emerging public health issue. Globally, the prevalence of excessive screen time among children ranged between 65% and 75% [3]. In developed countries such as Canada, the prevalence of children aged 3 years not meeting the recommended limit was as high as 94.7% [4]. In Malaysia, a national survey indicated that 52.2% of children aged <5 years exceeded screen use [5]. A recent study in the Petaling district of Selangor, Malaysia, showed that 91.4% of children aged <5 years exceeded the screen time limit [6]. The local prevalence was much higher than that in developing countries such as Brazil (28%) [7] and China (42.7%) [8]. Furthermore, it is likely that the recent movement control orders (MCOs) imposed following the COVID-19 pandemic would have led to further increase in screen time among children [9].

Despite some reported advantages of screen time when digital devices are used in moderation under parental guidance, excessive use beyond the recommended limits has been associated with multiple health risks, especially among young children. A positive association was detected between screen time with the child’s BMI *z* score and waist circumference [10], likely contributing to the increasing prevalence of obesity among Malaysian children from 11.9% in 2015 to 14.8% in 2019 [11]. In addition, other detrimental health outcomes such as sleep disturbances [12], conduct problems, hyperactivity [8], behavioral disorders [13], and poor quality of life [14] have also been reported. Although evidence-based guidelines have been established by the relevant stakeholders [2] and communicated to parents through the government child health record books, there is lack of strategies to assist parents in achieving the guidelines, resulting in a gap in parental knowledge, perception about screen time, and skills among parents.

### Previous Studies

During the early childhood period of preschool years, parents play the most fundamental role in shaping a child’s habits [15,16]. So far, only a handful of interventions targeting parents of preschoolers with the primary aim of screen time reduction have been established [17-22]. Moreover, almost all studies were conducted in Western countries, with a huge disparity in terms of parenting when compared with Asian cultures. Cross-cultural comparisons have highlighted the close connection between practically every element of parenting and the culture, including dietary practices; leisure-time pursuits; custom practices; and behaviors that they value, promote, reward, or disapprove to name a few [23,24]. As a multicultural country, there is a combination of various cultural and environmental influences that may affect local parenting practices and children’s screen use. The most commonly reported factors associated with screen time use included parental attitude toward screen time and low self-efficacy [25], both of which are amenable to health education intervention. Thus, screen time interventions for young children must incorporate both parents and children, as opposed to child-oriented interventions alone [18]. Previous studies have also indicated that parental control can result in the ability of preschoolers to self-regulate and subsequently reduce screen time [26]. However, these strategies have not been incorporated into previous studies. In addition, previous studies revealed that children from low socioeconomic backgrounds tend to have excessive screen time [27], partly owing to the lack of access to recreational areas and few opportunities to participate in after-school activities such as sports, music, and art [28]. Thus, there is a need to incorporate affordable alternative activities as an intervention toward reducing screen time among children from this target group. Furthermore, all previous screen time interventions targeted at preschoolers were conducted through face-to-face sessions. With the recent COVID-19 pandemic and the subsequent lockdown in many countries, coupled with multiple commitments of parents to adhere to face-to-face intervention, an alternative platform to explore the effectiveness of digital parental interventions to reduce children’s screen time is highly vital.

### Objectives

This study aimed to develop, implement, and evaluate the effectiveness of a parental digital health education intervention named *Stop and Play*, which was created using whiteboard animation videos based on the Social Cognitive Theory (SCT) by Bandura and delivered via WhatsApp (WhatsApp Inc). The intervention aimed to primarily reduce excessive screen time among preschoolers from low socioeconomic families in the Petaling district of Selangor, Malaysia. Secondary outcomes that were evaluated included the mother’s screen time knowledge, perception about the influence of screen time on the child’s well-being, self-efficacy to reduce screen time and increase physical activity, and screen time and the presence of digital devices in the child’s bedroom.

## Methods

### Study Design

**Figure 1:**
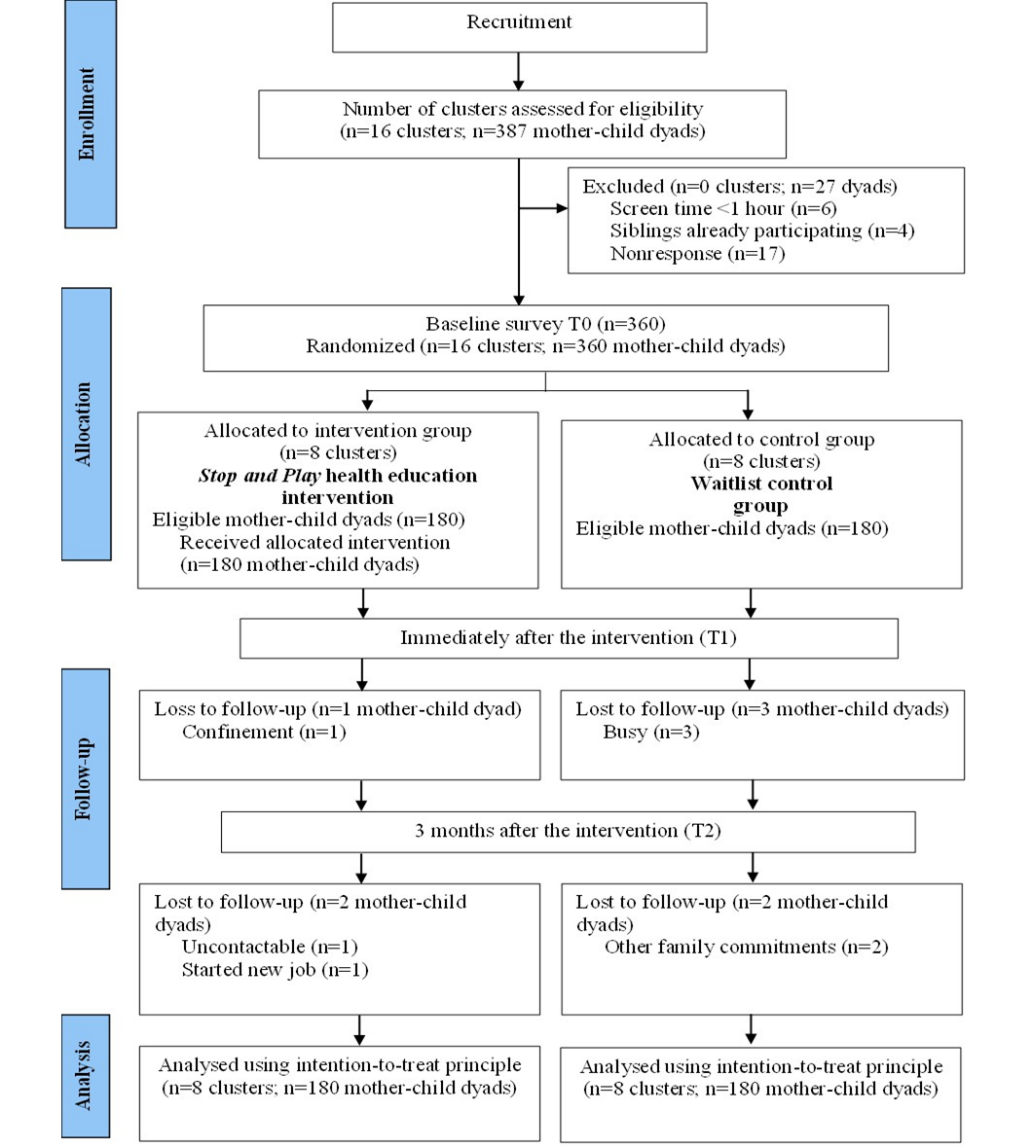
CONSORT (Consolidated Standards of Reporting Trials) diagram describing the recruitment and progress of participants throughout the study period. T0: baseline; T1: immediately after the intervention; T2: 3 months after the intervention.

This study used a 2-arm, parallel, cluster randomized controlled trial (RCT) design based on the CONSORT (Consolidated Standards of Reporting Trials) guideline’s extension for clustered RCTs. The clusters in this RCT were government preschools under the Community Development Department that provided education to children aged 3 to 4 years from low-income communities. All clusters were randomly assigned into the intervention group receiving the *Stop and Play* parental health education intervention or the waitlist control group using a 1:1 ratio. Figure 1 shows the CONSORT flow diagram of the recruitment and progress of all participants throughout the study period.

### Study Setting and Recruitment

Selangor is the most populated state in Malaysia, with the highest number of children (1.8 million children) [29]. This study was conducted in the Petaling district of Selangor, the district with the highest number of children below the age of 5 years. In addition, a recent study showed that 91.4% of children below the age of 5 years in Petaling had excessive screen time [6].

A total of 16 government preschools in the Petaling district were randomly selected. The operators of the preschools were then approached to participate in the study via phone calls and meetings. Recruitment of participants was originally planned for January 2020. However, owing to the closure of schools following COVID-19–related lockdowns, it was postponed until March 2021 to June 2021. The inclusion criteria for preschools were government preschools with classes for children aged 3 to 4 years. Regarding the mother-child dyad, those who were included were mothers with preschoolers aged 3 to 4 years, registered with government preschools in Petaling during the recruitment period in 2021 who reported their children’s screen time as >1 hour per day in the past month. They also needed to have access to smartphones and be willing to use WhatsApp as a medium of interaction. Mothers with physical or mental disabilities and children with physical disabilities, as certified by medical practitioners, were excluded from the study.

To recruit the participants, mothers of children aged 3 to 4 years within the designated clusters were identified from the enrollment database for the year 2021 and recruited. Preschool educators distributed electronic pamphlets containing brief information about the study to all mothers. Interested mothers can self-register using a Google Form link in the pamphlet. Eligible mothers who consented to participate were then added to a WhatsApp group managed by a research assistant. All participants were informed that they would receive the relevant interventions in stages within the next 6 months. Hence, they were blinded and unaware of their group allocation throughout the study.

### Sample Size Calculation

The sample size was calculated based on the primary outcome of detecting the mean screen time differences between the intervention group and control group. To detect a reduction of 43.2 (SD 105) minutes of screen time per day [30] with a 2-sided 5% significance level and 80% power, 100 parents were needed in each arm. After considering the design effect (intracorrelation coefficient [ICC]=0.01) [17] and attrition rate of 30% [21], a total of 350 participants were needed for both arms of the trial. As there were 135 eligible preschools in the Petaling district with a total enrollment of 5327 students, the number of clusters was determined by dividing the total enrollment by the sample size. Therefore, 16 clusters with equal sizes were considered for participant enrollment.

### Randomization and Blinding

Random sequence generation was conducted by a research assistant at the cluster level to avoid contamination. All eligible preschools were randomly allocated into the *Stop and Play* intervention group or the waitlist control group, with a 1:1 allocation ratio. The allocation was performed using a simple randomization technique via computer-generated software. Furthermore, a third-party assignment was applied to ensure the concealment of cluster allocation. Assignments for participants were enclosed in sequentially numbered opaque sealed envelopes. Enrollment of participants was performed by a second research assistant who was unaware of the cluster allocation. All participants within the clusters were invited to join the study. Following recruitment and baseline testing, a third research assistant invited all the parents assigned to the intervention arm to attend the intervention program. Thereafter, the intervention was administered by the researcher. Owing to the nature of the study, only single blinding was possible, whereby participating mothers were unaware of whether they have been allocated into the intervention group or control group.

### Intervention

As screen time was widely known to displace the time spent on physical activities, the term *Stop and Play* was coined for this intervention to reduce screen time (stop) and increase physical activity (play). It incorporates the 6 constructs under SCT, namely, knowledge, goal setting, self-efficacy, outcome expectation, observational learning, and problem-solving to improve parental knowledge and skills in reducing screen time among preschool children, by providing feasible activities that are culturally tailored as alternatives. The *Stop and Play* intervention also promoted self-directed play, a potential solution for parents who tend to use screen time as *digital babysitters*.

Following the COVID-19 pandemic, many health care professionals have strived to identify alternative evidence-based measures to deliver effective health education interventions at a time when face-to-face interactions were impossible. WhatsApp is an effective mode for delivering health education interventions [31]. Besides being compatible with any type of phone operating system, one of its main advantages is its ability to send messages swiftly [32]. It is also the most widely used mobile messenger app globally [33]. Furthermore, Malaysians are ranked as one of the world’s largest WhatsApp users [34].

The intervention module was developed through a process of consultation with a panel of health experts. To ensure its local cultural suitability, it was first pretested among 35 mother-child dyads of preschool children, who were not part of the main study. The delivery of the module was performed by the primary researcher, a physician. The intervention module took 4 weeks to be completed. Overall, 3 videos relevant to the educational content were created using whiteboard animation, and an additional video that portrayed success stories from other mothers was also included. A screenshot of the intervention is available in Multimedia Appendix 1. All videos were uploaded to YouTube and set to restricted viewing to avoid public access. The links to the YouTube videos were shared on a weekly basis with the participating mothers in the intervention group, with each video being approximately 3 minutes in duration. The videos were complemented with 2 infographic materials to reinforce the educational content. During the fourth week of intervention, there was a 12-hour problem-solving session, whereby the mothers could discuss the challenges they faced with the researcher via a private WhatsApp chat. On the basis of the viewing data on the YouTube channel, there was an average of 1 to 1.5 views per person per video. In addition, more than half of the participants in the intervention group (102/180, 56.7%) interacted with the researcher during the WhatsApp problem-solving session to obtain further assistance in reducing their child’s screen time. An average of 3 interactions per participant was recorded for this session. The total time taken to complete the intervention, including viewing the weekly videos and reviewing the infographics, was approximately 60 minutes over 4 weeks. A summary of the intervention module is presented in Multimedia Appendix 2. The intervention group also received a starter pack consisting of a program introduction, a *Stop and Play* fridge magnet, and a screen media diary for parents to report their child’s screen time. In contrast, the waitlist control group received the *Stop and Play* health education module only after the study was completed. Participants from both groups were given MYR 10 (US $2) as a token of appreciation upon successful completion of all questionnaires.

### Outcome

#### Primary Outcome Measure

The primary outcome of this study was the average screen time per day of the child, obtained using the SCREENS questionnaire [35]. The questionnaire showed good construct validity with good correlation (0.59-0.66) and moderate to good reliability (0.67-0.90). Mothers were asked to record their child’s screen time at home for a week before all 3 time points in the provided diary. During the baseline and immediate postintervention periods, all the children were staying entirely at home owing to MCO. However, at the third time point of data collection, the children had returned to schools, and no digital devices were allowed in the preschools. The preschools also did not use any digital devices for teaching purposes.

The use of four types of devices at home was considered as screen time: (1) television (including DVD, video games, PlayStation, and Xbox), (2) computers (desktop, laptop, and Chromebook), (3) telephones and smartphones, and (4) other mobile devices (tablets, iPad, and Nintendo Switch). For each device type, the weekday and weekend use times were averaged to obtain the device-specific screen time in hours per day, that is, ([total weekday × 5] + [total weekend day × 2]) / 7. The total screen time per day was calculated as the sum of use time for all 4 types of devices. To ensure the fidelity of the primary outcome in the questionnaire, the self-reported use time was cross-checked with screenshots of the screen media diary among 10% (18/180) of the participants. The request for the diary was made only after the completion of the final questionnaire. There was an agreement of 83% (15/18) between the reported time in the questionnaire and that in the diary.

#### Secondary Outcome Measures

##### Mother’s Knowledge

The items to measure parental knowledge were adapted from a previous study [36]. On the basis of the World Health Organization’s guidelines for screen time, sleep behavior, and physical activity, several other questions were added. The answers included “yes,” “no,” and “don’t know.” Correct answers were given 1 point, whereas wrong or uncertain answers were given 0 points. The total knowledge score ranged from 0 to 19. The internal consistency was reported as 0.77.

##### Mother’s Perception About the Influence of Screen Time on a Child’s Well-being

The total score for this outcome was calculated by averaging the responses to 11 items adapted from a previous study [37]. The items were classified into physical, cognitive, and social well-being on a 5-point Likert scale, ranging from 1=*strongly disagree* to 5=*strongly agree*. High scores represent high perception about the positive influence of screen time on their child’s well-being. The internal reliability for each of these constructs was 0.88, 0.90, and 0.72, respectively.

##### Mother’s Self-efficacy

Mother’s self-efficacy to reduce screen time was assessed using 9 items, 3 being adapted from the parenting self-efficacy scale used in the Infant Feeding Activity and Nutrition Trial [38], and another 6 were added by the researcher to better reflect parental belief on their ability to reduce screen time. The 6 additional items were “I am greatly confident I can: turn off mobile devices when eating at home, when eating at restaurants, provide alternative activities for my child in place of screens, be a role model by reducing my own leisure screen time, limit my child’s screen time to one hour per day, and ensure that my child does not have any screen time one hour before bedtime.” All responses were recorded on a 4-point Likert scale, ranging from 1=*not at all confident* to 4=*extremely confident*. The total scores were calculated by averaging the responses to the 9 items, with high scores indicating high self-efficacy to limit screen time. The internal consistency was 0.87.

In addition, mothers’ self-efficacy to increase their child’s physical activity was measured using 8 items adapted from a previous study [39]. The responses were recorded on a 4-point Likert scale, ranging from 1=*not at all confident* to 4=*extremely confident*. The total scores were calculated by averaging the responses to the 8 items, with high scores indicating high self-efficacy in increasing physical activity.

##### Mother’s Screen Time

Mothers’ weekday and weekend leisure screen time (excluding time spent on screen for work, school, or education purposes) was recorded in a way that is similar to recording the child’s screen time.

##### Physical Household Environment

Parents were also required to report if digital devices were present in the child’s bedroom (“yes”=1 point and “no”=0 point) [40]. A bedroom media score was computed by summing up the presence of 4 types of devices, as mentioned previously, in the child’s bedroom. Low scores indicated better physical home environment for screen time reduction. These items had good test-retest reliability, with ICC ranging from 0.51 to 0.96.

### Data Collection

Data collection was performed between March 2021 and December 2021. Web links to self-administered questionnaires on Google Forms were disseminated through WhatsApp at 3 time points, namely, baseline, immediate after the intervention, and 3 months after the intervention.

### Data Analysis

Statistical analysis was conducted using SPSS software (version 27.0; IBM Corp). Normality was assessed using skewness and kurtosis. Mean and SD were calculated for continuous data, whereas frequency and percentage were computed for categorical data. The generalized linear mixed model (GLMM) was used to evaluate the effect of the intervention after adjusting for covariates (mother’s age, mother’s education, combined household income, child’s sex, and baseline scores). The models were also adjusted for the clustering effect at the school level. GLMM was run for each outcome. Each model had school as the random effect, whereas the fixed effects that were included were group, time, and interaction between group and time. The group × time interaction effect was the primary variable of interest in each model. The 95% CI was set for mean estimation, with a *P* value of .05 as the level of significance. Bonferroni correction was applied to reduce any error rate, possibly resulting from the multiple numbers of tests performed. Data were analyzed using the intention-to-treat principle, whereby all participants were analyzed according to the initial group they were assigned to.

### Ethics Approval

This study was approved by the Ethics Committee for Research Involving Human Subjects at University Putra Malaysia (JKEUPM-2020-284). Web-based consent was obtained from both representatives of the clusters (preschools) and individual participating mothers before randomization. All participants were free to withdraw from the study at any given time for any given reason, and they would not be replaced. No personal information was requested through the WhatsApp group, and their privacy and confidentiality were protected throughout the study.

## Results

### Overview

The number of preschools and mother-child dyads participating in the *Stop and Play* trial is outlined in the CONSORT diagram (Figure 1). Following the 14-week recruitment period between March 2021 and June 2021, 16 preschools and 387 mother-child dyads were assessed for eligibility. All preschools (16/16, 100%) were deemed eligible, and their operators agreed to participate in this study. However, 2.3% (9/387) of the mother-child dyads were excluded as they did not meet the eligibility criteria. Of the 378 eligible participants, 360 (95.2%) mother-child dyads consented and completed the baseline questionnaire. The preschools were randomly assigned into the intervention and waitlist control groups, with each arm consisting of 50% (180/360) of the mother-child dyads. At the end of the study, the attrition rate was 1.7% (3/180) in the intervention group and 2.8% (5/180) in the waitlist control group.

### Comparisons of Sociodemographic Characteristics and Primary and Secondary Outcomes Between Intervention and Waitlist Control Groups at Baseline

The baseline characteristics of the participating mother-child dyads are presented in Table 1. Children in the intervention group were spending an average of 290.29 (SD 162.46) minutes on screen time per day as compared with average of 273.69 (SD 139.04) minutes per day in the control group. Overall, there was no significant difference between the sociodemographic characteristics and the primary and secondary outcomes between the intervention and control groups at baseline.

**Table 1:** Baseline characteristics of the intervention and waitlist control groups (N=360).

Characteristics			Intervention group (n=180, 50%)		Waitlist control group (n=180, 50%)		*t* test (*df*)^a^			Chi-square (df)			Mann-Whitney *U*			*P* value	
Mother’s age, mean (SD)			34.1 (4.3)		33.4 (4.7)		1.63 (358)			N/A^b^			N/A			.10	
**Mother’s highest education level, n (%)**								N/A			N/A			N/A			.30
	No formal education	0 (0)		1 (0.6)													
	Primary	6 (3.3)		4 (2.2)													
	Secondary	74 (41.1)		93 (51.7)													
	Diploma or certificate	59 (32.8)		52 (28.9)													
	Bachelor degree	39 (21.7)		28 (15.6)													
	Master degree or PhD	2 (1.1)		2 (1.1)													
Combined household income (n=357), median (IQR)			3000 (2000)		3300 (1975)		−0.06 (358)			N/A			N/A			.55	
**Employment status, n (%)**								N/A			3.8 (3)			N/A			.29
	Public	40 (22.2)		37 (20.6)													
	Private	50 (27.8)		67 (37.2)													
	Self-employed	9 (5)		8 (4.4)													
	Housewife or not working	81 (45)		68 (37.8)													
**Marital status, n (%)**								N/A			0.1 (1)			N/A			.76
	Married or living together	174 (96.7)		175 (97.2)													
	Divorced, widowed, separated, or single parent	6 (3.3)		5 (2.8)													
**Child’s age (years), n (%)**								N/A			0.1 (1)			N/A			.73
	3	53 (29.4)		56 (31.1)													
	4	127 (70.6)		124 (68.9)													
**Child’s sex, n (%)**								N/A			0.1 (1)			N/A			.75
	Male	99 (55)		96 (53.3)													
	Female	81 (45)		84 (46.7)													
**Ethnicity, n (%)**								N/A			3.4 (1)			N/A			.12
	Malay	166 (92.2)		174 (96.7)													
	Non-Malay	14 (7.8)		6 (3.3)													
**Child’s device ownership, n (%)**								N/A			0 (1)			N/A			.91
	Yes	57 (31.7)		56 (31.1)													
	No	123 (68.3)		124 (68.9)													
**Number of children, n (%)**								N/A			0.8 (1)			N/A			.38
	Single	24 (13.3)		30 (16.7)													
	Multiple	156 (86.7)		150 (83.3)													
**Child**																	
	Screen time, mean (SD)	290.3 (162.5)		273.7 (139.1)		1.04 (358)			N/A			N/A			.30		
**Parental**																	
	Knowledge, mean (SD)	8.5 (3.7)		8.2 (3.1)		0.91 (358)			N/A			N/A			.36		
	Perception, mean (SD)	2.7 (0.5)		2.7 (0.6)		1.52 (358)			N/A			N/A			.13		
	Self-efficacy to reduce screen time, median (IQR)	2 (0.55)		2.00 (0.52)		N/A			N/A			15,845.5			.72		
	Self-efficacy to increase physical activity, median (IQR)	1.25 (0.50)		1.38 (0.72)		N/A			N/A			15,060			.24		
	Screen time, mean (SD)	266.8 (112.9)		261.2 (108.8)		0.48 (358)			N/A			N/A			.63		
**Environmental**																	
	Presence of device in child’s bedroom, median (IQR)	0 (1)		0 (1)		N/A			N/A			16,025			.83		

^a^The *t* test was 2 tailed.

^b^N/A: not applicable.

### Effect of the Intervention Using Intention-to-Treat Principle

On the basis of the mixed model analysis in Table 2, at the end of the 3-month intervention, the children in the intervention group showed significantly reduced screen time by 202 minutes as compared with the waitlist control group (β=−202.29, 95% CI −224.48 to −180.10; *P*<.001). The ICC for the children’s screen time was 0.04.

In addition, the results from the main group effects in Table 3 show that, at the end of the intervention, significant reduction in screen time was observed among children in the intervention group compared with those in the waitlist control group (F_2,1068_=173.07; *P*<.001).

**Figure 2:**
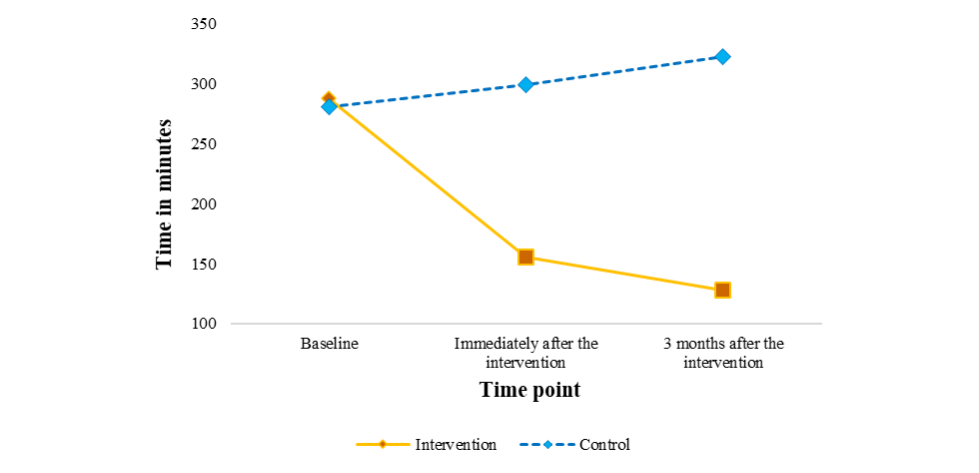
Plot of child’s screen time showing the interaction between intervention and waitlist control group at baseline, immediately after the intervention, and 3 months after the intervention.

Figure 2 shows the interaction of the child’s screen time between the intervention and waitlist control groups at baseline, immediately after the intervention, and 3 months after the intervention. There was significant group × time interaction for parental factors in the intervention group as compared with the waitlist control group. The mother’s knowledge score was significantly increased (β=6.88, 95% CI 6.11-7.65; *P*<.001). In contrast, the mother’s attitude toward screen time (β=−.26, 95% CI −0.38 to −0.15; *P*<.001) and perception about the influence of screen time on the child’s well-being had reduced (β=−.86, 95% CI −0.98 to −0.73; *P*<.001). In addition, there was improved self-efficacy among the mothers to reduce screen time (β=1.59, 95% CI 1.48-1.70; *P*<.001) and increase physical activity (β=.07, 95% CI 0.06-0.09; *P*<.001). Similarly, the mother’s leisure screen time also reduced (β=−70.43, 95% CI −91.51 to −49.35; *P*<.001), and the mother’s negative outcome expectations showed an increase (β=.34, 95% CI 0.18-0.50; *P*<.001). However, there was no significant postintervention effect on the presence of digital devices in the child’s bedroom.

**Table 2:** Intention-to-treat analysis of the effectiveness of Stop and Play intervention.

Variables and parameters		Adjusted β (SE; 95% CI)	*P* value^a^
**Child’s screen time**			
	Group × time	−202.23 (11.31; −224.48 to −180.10)	<.001^b^
**Mother’s knowledge**			
	Group × time	6.88 (0.39; 6.11 to 7.65)	<.001^b^
**Mother’s perception**			
	Group × time	−.86 (0.06; −0.98 to −0.73)	<.001^b^
**Self-efficacy to reduce screen time**			
	Group × time	1.59 (0.06; 1.48 to 1.70)	<.001^b^
**Self-efficacy to increase physical activity**			
	Group × time	.07 (0.01; 0.06 to 0.09)	<.001^b^
**Mother’s screen time**			
	Group × time	−70.43 (10.74; −91.51 to 49.35)	.001^b^
**Presence of screen device in child’s bedroom**			
	Group × time	−.03 (0.02; −0.08 to 0)	.08

^a^Using generalized linear mixed model adjusted for the mother’s age, mother’s education, household income, child’s sex, and baseline outcomes.

^b^Significant at *P*<.05.

**Table 3:** Fixed effects of the Stop and Play intervention on child’s screen time.

Variable	Parameter	*F* test (df)	*P* value^a^
Child’s screen time	Group	163.20 (1,1068)	<.001^b^
Child’s screen time	Group × time	173.07 (2,1068)	<.001^b^

^a^Using generalized linear mixed model adjusted for the mother’s age, mother’s education, household income, child’s sex, and baseline outcomes; the *t* test was 2 tailed.

^b^Significant at *P*<.05.

## Discussion

### Principal Findings

This trial investigated the effect of the *Stop and Play* health education intervention in reducing screen time among preschool children. It is an intervention developed based on SCT, using whiteboard animation videos, infographics reinforcement, and problem-solving session using the WhatsApp platform. Overall, the results indicated that it was successful in reducing preschoolers’ screen time and improving parental secondary outcomes.

On the basis of GLMM, significant reduction in children’s screen time was observed in the intervention group as compared with the waitlist control group. Our findings are consistent with previous studies [17,18,20]. However, the interventions in all these studies were delivered face to face before the COVID-19 pandemic. The favorable outcomes from our study show that a web-based intervention delivered remotely to mothers via WhatsApp can also reduce a child’s screen time. Hence, the use of digital technology in the delivery of interventions should be enhanced. Health care program developers involved in the planning and execution of health education interventions should take this into consideration, especially when the situation does not permit face-to-face interaction.

In this study, the baseline screen time reported among children was twice as high as that reported in previous studies [17,19-21], possibly attributed to the extra time spent at home as a result of MCO during the COVID-19 pandemic. Thus, it is encouraging to find that the *Stop and Play* intervention managed to reduce screen time by 202 minutes per day, which is much higher than the range (25-37 min/d) reported in other studies [17,18,20,21]. The vast difference could also be contributed by the students returning to their schools after MCOs were lifted during the third data collection period, where students have few opportunities to use screens. Nevertheless, in comparison with a previous study [18] that focused solely on children, our intervention targeted both mothers and children. Involving parents in goal setting, role modeling, and restructuring the physical environment has been associated with positive behavior changes among children [41]. A recent meta-analysis also reported that interventions that targeted parents of preschoolers had great effects in catalyzing behavior change [42].

Previously published studies of screen time reduction did not include any parental knowledge component, even though they might have been involved in the intervention. Increasing parental knowledge is the necessary first step for parents to initiate the subsequent actions to reduce their child’s screen time, such as restrictive viewing and supervision. In this study, the mother’s knowledge scores were significantly increased in the intervention group, indicating the importance of improving knowledge in interventions that aim for behavior change. However, improved knowledge alone is insufficient to produce screen time behavior change [43].

In this study, we also found that mothers’ perception about the influence of screen time on their child’s well-being had significant group and time interaction. Our findings were consistent with previous observations in which parental perception about the necessity to limit a child’s screen time resulted in significantly less screen time among preschool children [36]. However, the actual mean score of reduction was low. One of the possible barriers to achieving great mean reductions could be how certain parents perceived that screen time can improve their child’s language skills, as reported in a recent study [44]. When certain benefits of screen time for older children are portrayed in the media, parents tend to generalize the findings to young children also. Therefore, future interventions must emphasize that verbal and nonverbal communication is better formed through physical interaction than by acquiring the language through passive screen time, especially for young children aged <5 years [45].

Compared with a previous study, the *Stop and Play* intervention exerted a great impact on mothers’ self-efficacy to reduce screen time [21]. This could be attributed to a combination of factors. First, although the screen time recommendation was limited to 1 hour per day for children in this age group, the mothers were encouraged to set realistic and specific goals. This modification is necessary to boost their belief that screen time reduction would be achievable for their children. In addition, they were given practical advice on how to solve real-life situations that might involve the need to give more screen time to the children, for example, when waiting for meals at restaurants. Alternative bedtime routines other than screens such as saying family prayers were also put forth as suggestions. Mothers from low socioeconomic backgrounds can consider using recyclable items or regular household items as affordable alternative activities. According to the feedback provided by the mothers in this study, the main activities that successfully displaced their child’s screen time were indoor bowling; art and craft activities such as homemade play dough; cardbox houses for pretend play, built from recyclable materials; and free play using household materials.

A compelling finding from a recent study showed that Malaysian parents found it difficult to incorporate the reading culture into the daily lives of children [46]. In addition, as many as one-third of urban poor households did not have any suitable reading materials such as books for children [47]. Hence, despite the strong evidence in previous studies that promoted books as a substitute for screen time, it could not be adapted in our study. In contrast, a salient component of the intervention in this study was the introduction to self-directed play. Mothers were encouraged to cultivate independent play for the preschoolers in a safe and prepared environment. Besides stimulating imagination and creativity, independent play can also give the mother a much-needed break to focus on other household chores. In addition, the video sharing of success stories from other mothers could motivate mothers’ self-efficacy. Positive findings further support the incorporation of SCT constructs into interventions aimed at reducing children’s screen time.

Next, the self-efficacy to increase physical activity among mothers was significantly high in the intervention group, albeit with just minor increase in mean scores. According to the observational learning construct, children are highly influenced by their parents’ uptake of physical activity. Thus, future interventions should promote physical activity for the whole family as compared with focusing solely on children. The minor increase in mean scores could be explained by the challenges for mothers to engage their children in physical activity outside the house owing to MCO during the pandemic. A systematic review found significant increase in self-efficacy to increase physical activity when interventions were conducted face to face and objective measures of physical activity accompanied by self-monitoring and performance feedback were reported [48]. Although it was initially planned, the objective measures of physical activity in this study could not be obtained owing to the limitations of the pandemic.

In addition, mothers in the intervention group showed significant reduction in leisure screen time by 70 minutes per day. Although most studies of child’s screen time stressed the importance of reducing parental screen time, there is a lack of RCTs that examine the actual reduction. Nevertheless, an RCT using mobile technology to control screen time among adults reported large reduction of 130 minutes within 2 weeks of intervention [49]. This could be attributed to the specific screen time reduction strategies targeted at adults, while emphasizing the health implication such as noncommunicable diseases from sedentary lifestyles following prolonged screen time behavior. As such, health care professionals and other relevant stakeholders should promote screen time reduction as beneficial for both the child and the parents alike.

In addition, the presence of digital devices in the child’s bedroom has been strongly associated with excessive screen time [50]. Nevertheless, this intervention did not demonstrate any reduction in the presence of digital devices in the child’s bedroom. This finding can be attributed to the cultural and economic background of the study population. Families with low socioeconomic status in urban areas of Malaysia typically live in low-cost flats with limited space and have low number of devices per family member. Thus, it is highly conceivable that screen devices such as televisions would be placed in common spaces such as the living room rather than the bedroom. In this study, approximately 68.6% (247/360) of the participating children did not own any personal digital devices that they could take into their bedrooms. Moreover, bed-sharing with parents is a common practice in Malaysia compared with Western culture, as shown in a recent study that reported high rate of bed-sharing practice among Malaysian mothers with their children [51]. In other words, most children probably did not have a bedroom of their own at the age of 3 to 4 years. These results highlighted the contrasting findings between different cultural and socioeconomic settings that warrant further exploration.

### Strengths and Limitations

This is one of the pioneer studies that provided strong evidence of the effectiveness of a digitally delivered screen time reduction intervention for parents of preschool children. Furthermore, the intervention also measured self-efficacy as a principal construct of the SCT, thus affirming its use in screen time studies. Low attrition rates indicated strong interest among participants in the reduction of screen time among their children. Furthermore, the use of whiteboard animation videos for health education resonates with young mothers who were mainly millennials, that is, Generation Z. Moreover, the remote method of delivery allowed mothers to conveniently access the intervention at a time and place suitable for them.

Nevertheless, this study has few limitations. First, the findings need to be interpreted with caution owing to the limited generalizability. Although the use of screen media diaries helped to minimize recall bias, self-reporting by mothers could have been influenced by social desirability related to underreporting their child’s screen time and overembellishing their parenting capabilities, thus raising issues about the fidelity of the outcomes. Second, owing to the remote method of health education delivery, the lack of physical interaction could have resulted in poor interpersonal relationship between the researcher and the participants, indirectly affecting the strength of the outcomes. Furthermore, we were unable to measure the exact amount of physical activity because outdoor activities were not possible during the pandemic. In addition, data collection for both postintervention time points was also subjected to different conditions as compared with that at the baseline owing to the closure of schools during MCO. During the baseline period and 1-month data collection period, children were undergoing web-based school sessions for 1 hour per day, thus leading to long period of participation in screen-based activities at home. However, during the 3-month postintervention data collection period, physical lessons had resumed for all schools, leaving the children with less time to spend on screen-based activities. Finally, we were unable to explicitly verify whether all mothers watched the given videos, as the number of views could have represented repeated viewing from the same participants.

### Conclusions

*Stop and Play*, a theory-based digital health education intervention for parents, was found to effectively reduce preschoolers’ screen time and improve parent-related secondary outcomes. Evidence from this study supports the idea of using digital technology as a mode of delivery for screen time reduction interventions for parents. In addition, the digital method is also a viable option for parents who are unable to be present physically for the intervention sessions. Besides contributing to the growing body of literature, our study also reveals new insights into screen time reduction interventions from the perspective of parents from a non-Western culture in a developing country.

Considering the proven benefits of screen time reduction, this intervention should be implemented in the primary health care setting to educate parents and be incorporated into the child health wellness programs in preschools and daycare facilities. Nevertheless, proper planning in terms of human resource availability and training must be considered before the full implementation of the intervention. Furthermore, future studies should also be expanded to other study populations from middle or high socioeconomic backgrounds. It is also recommended for future studies to apply mediation analysis to investigate the extent to which the changes in a child’s screen time can be attributed to changes within the secondary outcomes. More importantly, the sustainability of this intervention must be ascertained by evaluating the effectiveness of interventions at long intervals.

## Appendix

Multimedia Appendix 1 Sample of Stop and Play whiteboard animation screen capture.

Multimedia Appendix 2 Summary of Stop and Play screen time intervention module.

Multimedia Appendix 3 CONSORT-eHEALTH checklist (V 1.6.2).

## Abbreviations

CONSORT: Consolidated Standards of Reporting Trials
GLMM: generalized linear mixed model
ICC: intracorrelation coefficient
MCO: movement control order
RCT: randomized controlled trial
SCT: Social Cognitive Theory

## Data Availability

The data sets generated and analyzed during this study are available from the corresponding author upon reasonable request.
