# Supplementary figures and images for: Stop and Play Digital Health Education Intervention for Reducing Excessive Screen Time Among Preschoolers From Low Socioeconomic Families: Cluster Randomized Controlled Trial
Source: J Med Internet Res. 2023 May 4;25:e40955. doi: 10.2196/40955 (PMC10196888; doi:10.2196/40955)

Multimedia Appendix 1. Sample of *Stop and Play* whiteboard animation screen capture.


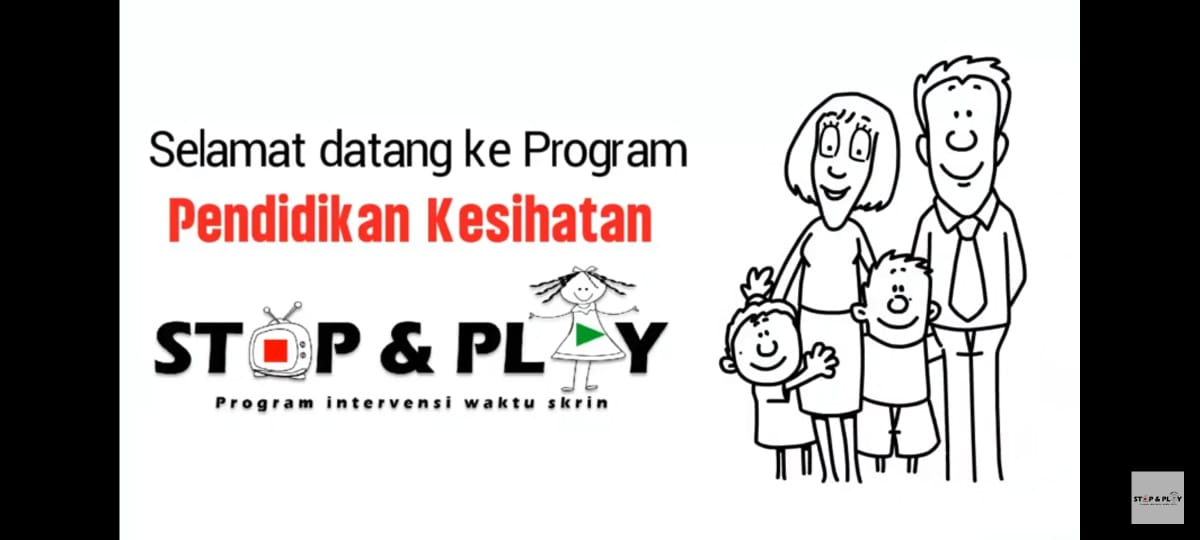

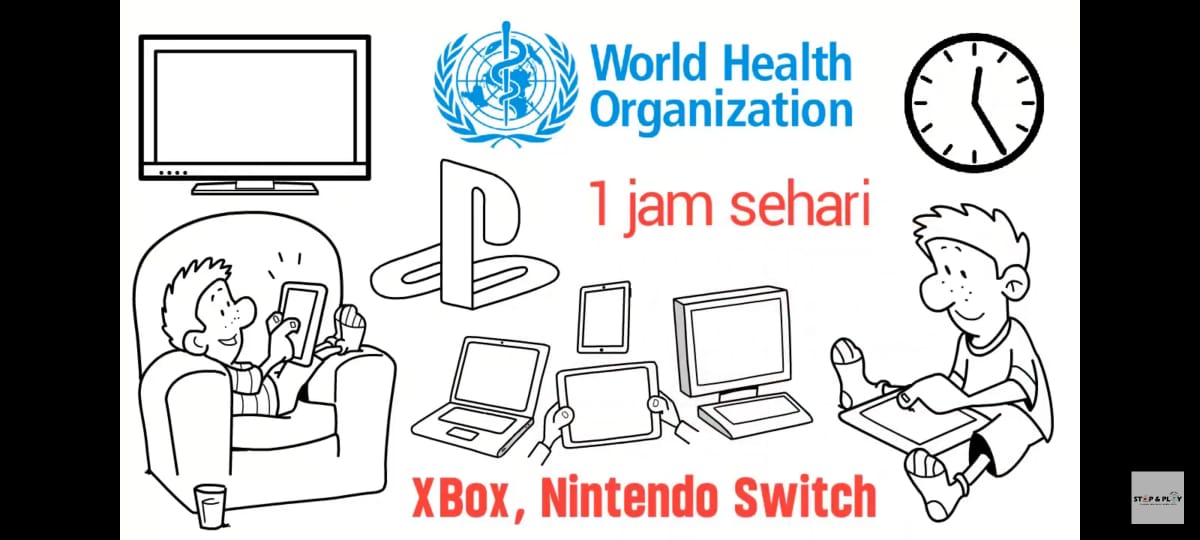

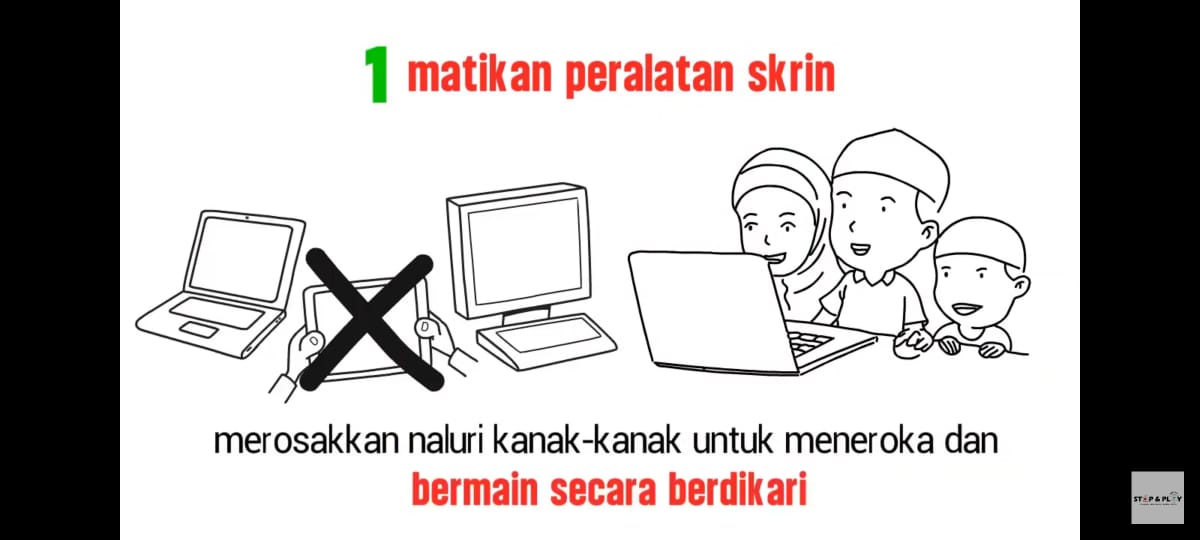

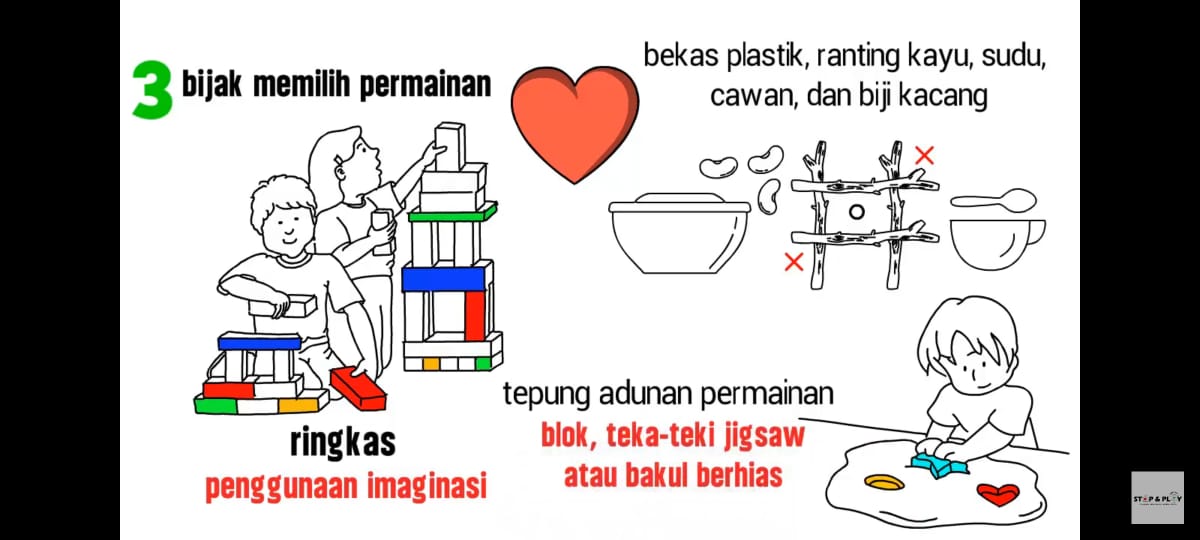

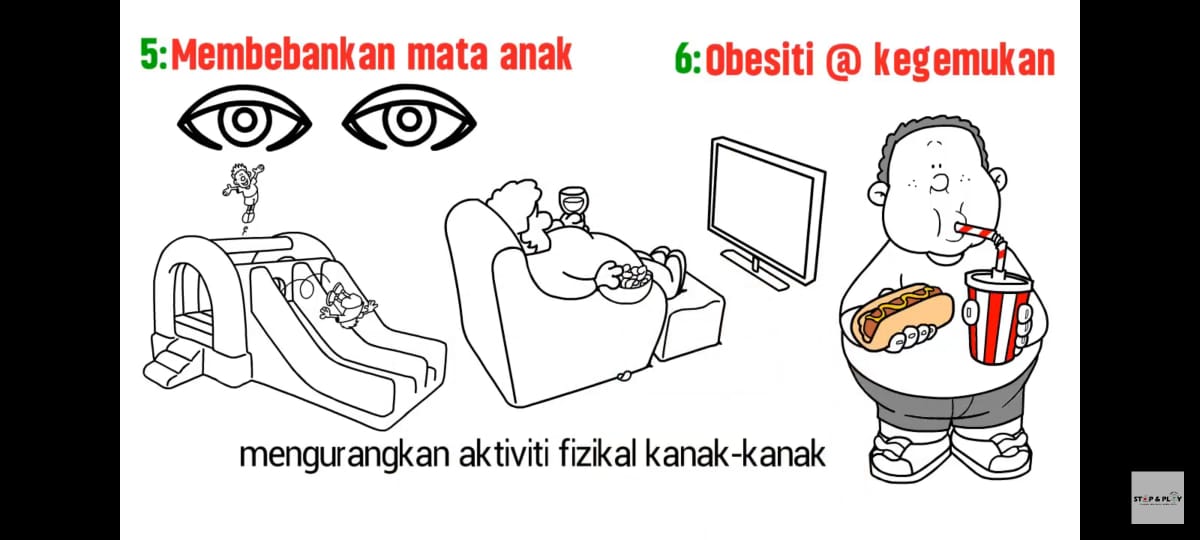

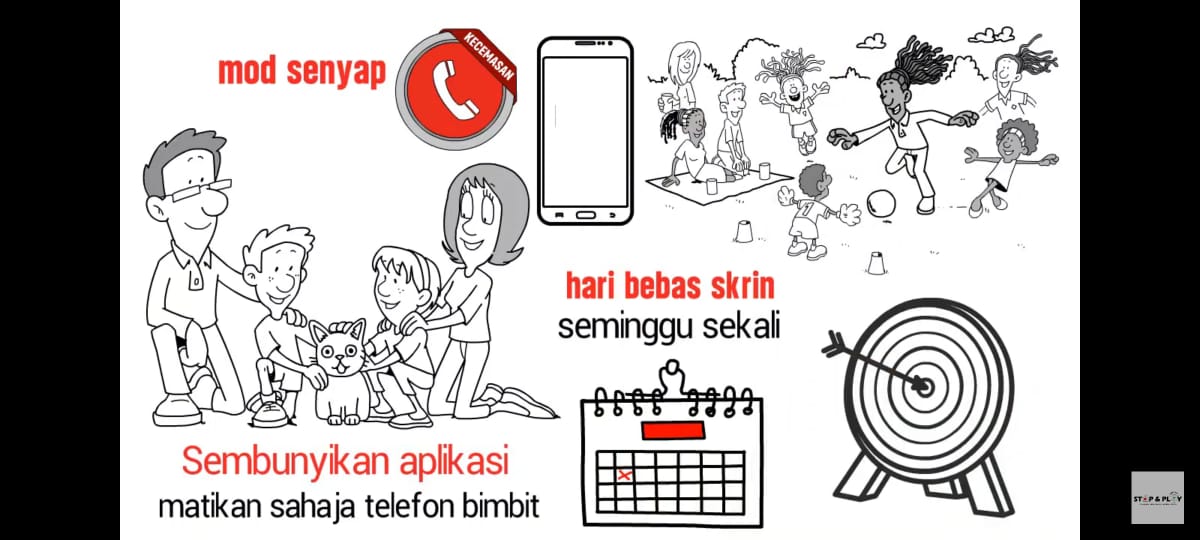

Supplement: Multimedia Appendix 1 [file jmir_v25i1e40955_app1.docx]
