# Supplementary material for: Stop and Play Digital Health Education Intervention for Reducing Excessive Screen Time Among Preschoolers From Low Socioeconomic Families: Cluster Randomized Controlled Trial
Source: J Med Internet Res. 2023 May 4;25:e40955. doi: 10.2196/40955 (PMC10196888; doi:10.2196/40955)
Supplement: Multimedia Appendix 2 [file jmir_v25i1e40955_app2.docx]

Multimedia Appendix 2. Summary of *Stop and Play* screen time intervention module

| **Week** | **Mode of delivery** | **Topic** | **SCT constructs** |
| --- | --- | --- | --- |
| 1 | Printed material | Introduction to *Stop and Play* health education intervention. |  |
|  | Video 1 | Screen time definition, recommendations & negative implications. | Knowledge, outcome expectations. |
|  |  | Parents as role model. | Observational learning |
|  |  | Screen time related goals. | Goal setting |
|  | Fridge Magnet | Screen time goal of 1 hour. | Goal setting |
|  | Infographics | Detailed examples of incremental goal setting. | Goal setting |
|  |  | Impact of screen time on child’s well-being. | Outcome expectations, knowledge. |
| 2 | Video 2 | Setting screen time rules. | Self-efficacy, Goal setting, outcome expectations |
|  |  | Healthy bedtime routines, removal of gadgets from child’s bedroom, recommended  sleep duration, effects of screen on child’s sleep. | Knowledge, outcome expectation |
|  |  | Types of moderate to vigorous physical activity, recommended physical activity duration and effects of screen on child’s physical activity. | Knowledge, outcome expectation |
|  | Infographics | Positive parenting: Connection before correction. | Knowledge, Self-efficacy |
|  |  | WHO guidelines on recommended screen time, physical activity & sleep duration. | Knowledge |
| 3 | Video 3 | Self-directed play. | Knowledge, self-efficacy |
|  |  | Believe to achieve, rising above your challenges. | Self-efficacy |
|  |  | Culturally suited alternative activities. | Knowledge, self-efficacy |
|  | Infographics | You are Key to your success. | Self-efficacy |
|  |  | Alternative activities to screen time. | Knowledge, self-efficacy |
| 4 | WhatsApp consultation | Problem Solving. | Problem solving |
|  | Video 4 | Success Stories. | Observational learning, self-efficacy |
