# Supplementary material for: Stop and Play Digital Health Education Intervention for Reducing Excessive Screen Time Among Preschoolers From Low Socioeconomic Families: Cluster Randomized Controlled Trial
Source: J Med Internet Res. 2023 May 4;25:e40955. doi: 10.2196/40955 (PMC10196888; doi:10.2196/40955)
Supplement: Multimedia Appendix 3 [file jmir_v25i1e40955_app3.pdf]

CONSORT-EHEALTH Checklist V1.6.2 Report	Manuscript Number	40955

(based on CONSORT-EHEALTH V1.6), available at [http://tinyurl.com/consort-ehealth-v1-6].

Date completed

1/9/2023 13:10:40

by

Diana Raj

Stop & Play digital health education intervention in reducing excessive screen time among preschoolers from low socioeconomic families in Malaysia: A Cluster Randomized Control Trial

TITLE

1a-i) Identify the mode of delivery in the title

Yes "Stop & Play' digital health education intervention.

1a-ii) Non-web-based components or important co-interventions in title

Not applicable. "All intervention components were web-based."

1a-iii) Primary condition or target group in the title

Yes. "reducing excessive screen time among preschoolers from low socioeconomic families in Malaysia."

ABSTRACT

1b-i) Key features/functionalities/components of the intervention and comparator in the METHODS section of the ABSTRACT

Yes. "Single-blind two-arm, cluster randomized control trial was conducted among 360 mother-child dyads attending government preschools in Petaling district who were randomly allocated into the intervention and wait list control groups". "using social cognitive theory

1b-ii) Level of human involvement in the METHODS section of the ABSTRACT

"This 4-week intervention developed using whiteboard animation videos, infographics, and a problem-solving session was delivered via WhatsApp".

1b-iii) Open vs. closed, web-based (self-assessment) vs. face-to-face assessments in the METHODS section of the ABSTRACT

Yes. It was purely web based with no face to face sessions. "Single-blind two-arm". "Delivered via WhatsApp." "self-administered questionnaires administered."

1b-iv) RESULTS section in abstract must contain use data

Yes. "Total of 352 dyads completed the study, 177 in the intervention group and 175 in the waitlist control group with attrition rate of 2.2% (8/360)."

1b-v) CONCLUSIONS/DISCUSSION in abstract for negative trials

We strongly believe in the importance of reporting negative trials. However, the primary outcome in our study showed positive results.

INTRODUCTION

2a-i) Problem and the type of system/solution

Yes. "Globally, the prevalence of excessive screen time among children ranged between 65%-75% [3]."In Malaysia, a national survey indicated that 52.2% of children under the age of five years old exceeded screen usage [5]. "The local prevalence was much higher than in developing countries such as Brazil (28%) [7] and China (42.7%) [8]." "there was lack of strategies to assist parents in achieving the guidelines resulting in a gap in parental knowledge, perception on screen time and skills among parents." "almost all were conducted in Western countries with a huge disparity in terms of parenting when compared with Asian cultures." "screen time interventions for young children must incorporate both parents and children as opposed to child-oriented interventions alone" "there is a need to incorporate affordable alternative activities "all previous screen time interventions targeted at preschoolers were conducted through face-to-face sessions." "alternative platform to explore the effectiveness of digital parental interventions"

2a-ii) Scientific background, rationale: What is known about the (type of) system

Yes. "In Malaysia, 52.2% of children under the age of five had too much screen usage (5). This prevalence was higher compared to other developing countries such as Brazil (28%) (6) and China (42.7%) (7)."Although evidence based guidelines have been outlined (2), the tools and strategies to achieve the guidelines remain unclear to parents struggling with digital parenting." "all previous screen time interventions for preschoolers were conducted through face-to-face sessions in developed countries and impractical to apply especially during the pandemic."

Does your paper address CONSORT subitem 2b?

Yes. "This study aimed to develop, implement, and evaluate the effectiveness of a social cognitive theory-based; Stop & Play digital health education intervention created using whiteboard animation videos and delivered via WhatsApp to primarily reduce preschoolers excessive screen time among low socioeconomic mothers in Petaling district Selangor, Malaysia. Secondary outcomes were improvements in mothers screen time knowledge, perception of screen time on child's wellbeing, self-efficacy to reduce screen time and increase physical activity, mothers screen time, and presence of screen in child's bedroom."

METHODS

3a) CONSORT: Description of trial design (such as parallel, factorial) including allocation ratio

Yes. "This study used a two-arm parallel, cluster randomized control trial (RCT);" "using a 1:1 ratio."

3b) CONSORT: Important changes to methods after trial commencement (such as eligibility criteria), with reasons

No changes were made after trial commencement.

3b-i) Bug fixes, Downtimes, Content Changes

Not applicable in our study.

4a) CONSORT: Eligibility criteria for participants

Yes. "The inclusion criteria for preschools were government preschools with classes for children aged 3-4 years. As for the mother-child dyad, those who were included were mothers with preschoolers aged 3-4 years old registered with government preschools in Petaling during the recruitment period in 2021 who reported their children's screen time as more than an hour per day in the past month. They also needed to have access to smartphones and be willing to use WhatsApp as a medium of interaction. Mothers with physical or mental disabilities and children with physical disabilities as certified by medical practitioners were excluded from the study".

4a-i) Computer / Internet literacy

Yes. "Eligibility include mothers who had smart phones and willing to use WhatsApp as a medium of interaction."

4a-ii) Open vs. closed, web-based vs. face-to-face assessments:

"Sixteen government preschools within the Petaling district were randomly selected using random number allocation. They were then approached to participate in the study through phone call and meeting with the district head of preschools. Recruitment of participants was originally planned to be carried out in January 2020, however due to the closure of schools and COVID-19 related lockdowns, recruitment took place through the preschools from March-June 2021."

4a-iii) Information giving during recruitment

Yes." All participants were informed that they would receive the relevant interventions in stages within the next six months. Hence, they were blinded and unaware of their group allocation throughout the study".

4b) CONSORT: Settings and locations where the data were collected

Yes. "This study was conducted in the Petaling district of Selangor, the district with the highest number of children under five years of age".

4b-i) Report if outcomes were (self-)assessed through online questionnaires

Yes. "Online links to self-administered questionnaires on Google forms were disseminated through WhatsApp at three time points namely baseline, immediate post-intervention, and 3-month post-intervention."

4b-ii) Report how institutional affiliations are displayed

Yes. The identification of institutional affiliation was displayed in the questionnaire and intervention materials.

5) CONSORT: Describe the interventions for each group with sufficient details to allow replication, including how and when they were actually administered

5-i) Mention names, credential, affiliations of the developers, sponsors, and owners

Not applicable as we used social media i.e. WhatsApp.

5-ii) Describe the history/development process

Yes. "The intervention module was developed through a process of consultation with a panel of health experts. To ensure its local cultural suitability, it was first pre-tested among 35 mother-child dyads of preschool children who were not part of the main study. The delivery of the module was performed by the primary researcher, a medical doctor."

5-iii) Revisions and updating

The health intervention module used in this study is the first version.

5-iv) Quality assurance methods

Yes. Validated self-administered questionnaires administered." " The intervention module was pilot tested among 35 mother-child dyads of preschool children who were not part of the main study"

5-v) Ensure replicability by publishing the source code, and/or providing screenshots/screen-capture video, and/or providing flowcharts of the algorithms used

Details of the content of the intervention module is described in the intervention section and in Multimedia Appendix 2. Screen capture video is also provided in Multimedia Appendix 1 to ensure replicability.

5-vi) Digital preservation

Not applicable to this study

**5-vii) Access**		
YEs. "Interested mothers can self-register using a Google form link in the pamphlet. Eligible mothers who consented to participate were then added to a WhatsApp group managed by a research assistant. " " Three videos relevant to the educational content were created using whiteboard animation and an additional video that portrayed success stories from other mothers was also included. All videos were uploaded to YouTube and set as unlisted viewing to avoid public access. The links to the YouTube videos were shared on a weekly basis with the participating mothers in the intervention group with each video being approximately three minutes. The videos were complemented with two infographic materials to reinforce the educational content. During the fourth week of intervention, there was a 12-hour problem-solving session whereby the mothers can discuss the challenges they faced with the researcher via a private WhatsApp chat."		
**5-viii) Mode of delivery, features/functionalities/components of the intervention and comparator, and the theoretical framework**		
Yes. This 4-week intervention using newly developed whiteboard animation videos, weekly reinforcement infographics and a problem -solving session was delivered by researcher via WhatsApp. Videos pertaining the content was created using whiteboard animation, uploaded to YouTube with links provided to participants. "It incorporates six constructs namely knowledge, goal setting, self-efficacy, outcome expectation, observational learning and problem solving from Bandura's social cognitive theory to improve parental knowledge, and skills in reducing screen time among preschool children whilst providing feasible alternative activities that are culturally tailored to support the reduction. It also promotes self-directed play to provide a viable solution for parents who tend to use screens as digital babysitters." In depth description of the content was described in the intervention section and Multimedia Appendix 2.		
**5-ix) Describe use parameters**		
Yes. "each video being approximately three minutes." " The total time taken to complete the intervention including viewing the weekly videos and reviewing the infographics was approximately 60 minutes over four weeks." Questionnaires took 15-20 minutes to be filled at each timepoint.		
**5-x) Clarify the level of human involvement**		
Yes. "The delivery of the module was performed by the primary researcher, a medical doctor". During the fourth week of intervention, there was a 12-hour problem-solving session whereby the mothers can discuss the challenges they faced with the researcher via a private WhatsApp chat."		
**5-xi) Report any prompts/reminders used**		
Yes. Following recruitment and baseline testing, a third research assistant invited parents under the intervention arm to attend the intervention program. "Links to the YouTube channel was given to participating mothers in the intervention group weekly over the course of four weeks respectively. This was followed by two infographic materials to further reinforce the video content."		
**5-xii) Describe any co-interventions (incl. training/support)**		
This is not applicable in our study.		
**6a) CONSORT: Completely defined pre-specified primary and secondary outcome measures, including how and when they were assessed**		
Yes. "Primary outcome was the child's screen time whilst secondary outcomes included mother's screen time knowledge, perception of screen time on the child's wellbeing, self-efficacy to reduce the child's screen time and increase physical activity, mother's screen time, and presence of screen device in child' s bedroom. Validated self-administered questionnaires were administered at baseline, immediately, and 3-months post-intervention".		
**6a-i) Online questionnaires: describe if they were validated for online use and apply CHERRIES items to describe how the questionnaires were designed/deployed**		
Not applicable. Data collection was done using Google forms at baseline, one month and 3-month post intervention due to movement control orders during the pandemic.		
**6a-ii) Describe whether and how "use" (including intensity of use/dosage) was defined/measured/monitored**		
Outcomes were assessed " through WhatsApp at three time points namely baseline, immediate post-intervention, and 3-month post-intervention."		
**6a-iii) Describe whether, how, and when qualitative feedback from participants was obtained**		
Qualitative feedback was only obtained during the pilot testing phase and not during the main trial.		
**6b) CONSORT: Any changes to trial outcomes after the trial commenced, with reasons**		
Yes. "This study was conducted in the Petaling district of Selangor, the district with the highest number of children under five years of age".		
**7a) CONSORT: How sample size was determined**		
**7a-i) Describe whether and how expected attrition was taken into account when calculating the sample size**		
Yes. "The sample size was calculated based on the primary outcome of detecting mean screen time differences between the intervention group and control group. To detect a reduction of 43.2 (SD 105.00) minutes of screen time per day [30] with a two-sided 5% significance level and 80% power, 100 parents were needed in each arm respectively. After considering the design effect (Intra-correlation coefficient, ICC =0.01) [17] and an attrition rate of 30% [21], a total of 350 participants were needed for both arms of the trial."		
**7b) CONSORT: When applicable, explanation of any interim analyses and stopping guidelines**		
Yes. "Primary outcome was the child's screen time whilst secondary outcomes included mother's screen time knowledge, perception of screen time on the child's wellbeing, self-efficacy to reduce the child's screen time and increase physical activity, mother's screen time, and presence of screen device in child' s bedroom. Validated self-administered questionnaires were administered at baseline, immediately, and 3-months post-intervention".		
**8a) CONSORT: Method used to generate the random allocation sequence**		
Yes. "All eligible preschools were randomly allocated into the Stop & Play intervention group or the waitlist control group with a 1:1 allocation ratio. The allocation was performed using a simple randomization technique via computer-generated software."		
**8b) CONSORT: Type of randomisation; details of any restriction (such as blocking and block size)**		
Yes. All eligible preschools were randomly allocated into the Stop & Play intervention group or the waitlist control group with a 1:1 allocation ratio. The allocation was performed using a simple randomization technique via computer-generated software. No blocking was used.		
**9) CONSORT: Mechanism used to implement the random allocation sequence (such as sequentially numbered containers), describing any steps taken to conceal the sequence until interventions were assigned**		
Yes. "Furthermore, a "third-party" assignment was applied to ensure the concealment of cluster allocation. Assignments for participants were enclosed in sequentially numbered opaque sealed envelopes."		
**10) CONSORT: Who generated the random allocation sequence, who enrolled participants, and who assigned participants to interventions**		
Yes. Three separate research assistants. "External involvement using "third-party" assignment was used to ensure cluster allocation concealment. Assignments were enclosed in sequentially numbered opaque sealed envelopes. Enrolment of participants was performed by a second research assistant who was masked to the cluster allocation. All participants within the clusters were invited to join the study. Following recruitment and baseline testing, a third research assistant invited parents under the intervention arm to attend the intervention program."		
**11a) CONSORT: Blinding - If done, who was blinded after assignment to interventions (for example, participants, care providers, those assessing outcomes) and how**		
**11a-i) Specify who was blinded, and who wasn't**		
Yes. "Due to the nature of the study, only single blinding was possible whereby participating mothers were unaware of whether they have been allocated into the intervention or control group."		
**11a-ii) Discuss e.g., whether participants knew which intervention was the "intervention of interest" and which one was the "comparator"**		
Yes. "participating mothers were unaware of whether they have been allocated into the intervention or control group." As comparator group was a waitlist control group, the only difference between the groups was the timing of intervention whereby the control group received the exact same intervention once the trial was completed.		
**11b) CONSORT: If relevant, description of the similarity of interventions**		
Not relevant to our study.		
**12a) CONSORT: Statistical methods used to compare groups for primary and secondary outcomes**		
Yes. "The Generalized Linear Mixed Model (GLMM) was used to evaluate the effect of the intervention after adjusting for covariates (mother's age, mothers' education, combined household income, child's sex, and baseline scores). The models were also adjusted for the clustering effect at the school level."		
**12a-i) Imputation techniques to deal with attrition / missing values**		
We did not impute any data as attrition was minimal and advanced statistical methods were used.		
**12b) CONSORT: Methods for additional analyses, such as subgroup analyses and adjusted analyses**		
Generalized linear mixed model was used to evaluate the effect of the intervention, adjusting for covariates (mother's age, mothers' education, combined household income, child's sex and baseline scores).		
RESULTS		
**13a) CONSORT: For each group, the numbers of participants who were randomly assigned, received intended treatment, and were analysed for the primary outcome**		
Yes. "Of the eligible participants, 360 mother-child dyads consented and completed the baseline questionnaire. The preschools were randomly assigned into the intervention and waitlist control group, with each arm consisting of 180 mother-child dyads." Data was analyzed using the intention to treat principal whereby participants were analyzed according to the initial assigned group.		
**13b) CONSORT: For each group, losses and exclusions after randomisation, together with reasons**		
"At the end of the study, the attrition rate was 1.7% (3/180) in the intervention group and 2.8% (5/180) in the waitlist control group." Reasons for attrition are provided in Figure 1.		
**13b-i) Attrition diagram**		
Yes. "Figure 1: CONSORT diagram describing the recruitment and progress of participants throughout the study period."		
**14a) CONSORT: Dates defining the periods of recruitment and follow-up**		
Yes. :"Following the 14-weeks recruitment period between March and June 2021" " Data collection was undertaken between March and December 2021. Online links to self-administered questionnaires on Google forms were disseminated through WhatsApp at three time points namely baseline, immediate post-intervention, and 3-month post-intervention."		
**14a-i) Indicate if critical "secular events" fell into the study period**		

Yes. "Recruitment of participants was originally planned for January 2020. However, due to the closure of schools following COVID-19-related lockdowns, it was postponed until March-June 2021."		
**14b) CONSORT: Why the trial ended or was stopped (early)**		
Not applicable. The trial continued during the pandemic		
**15) CONSORT: A table showing baseline demographic and clinical characteristics for each group**		
Yes. Baseline demographics are depicted in Table 1.		
**15-i) Report demographics associated with digital divide issues**		
Yes. "Overall, there was no significant difference between the sociodemographic characteristics as well as the primary and secondary outcomes between the intervention and control groups at baseline."		
**16a) CONSORT: For each group, number of participants (denominator) included in each analysis and whether the analysis was by original assigned groups**		
**16-i) Report multiple "denominators" and provide definitions**		
Yes. ""Figure 1 shows the final number of participants in each group."<br>"Data was analyzed using the intention to treat principal whereby participants were analyzed according to the initial assigned group".		
**16-ii) Primary analysis should be intent-to-treat**		
"Data was analyzed using the intention to treat principal whereby participants were analyzed according to the initial assigned group".		
**17a) CONSORT: For each primary and secondary outcome, results for each group, and the estimated effect size and its precision (such as 95% confidence interval)**		
Yes. At the end of the 3-months, children in the intervention group had significantly reduced their screen time by 202 minutes as compared to the waitlist control group (β=-202.29, 95% CI -224.48 to -180.10, P<.001). Results for secondary outcomes are outlined in Table 2.		
**17a-i) Presentation of process outcomes such as metrics of use and intensity of use**		
Not applicable to our study.		
**17b) CONSORT: For binary outcomes, presentation of both absolute and relative effect sizes is recommended**		
All outcomes were in continuous variables.		
**18) CONSORT: Results of any other analyses performed, including subgroup analyses and adjusted analyses, distinguishing pre-specified from exploratory**		
Yes. Results were adjusted for covariates.		
**18-i) Subgroup analysis of comparing only users**		
Subgroup analysis was not performed in this study.		
**19) CONSORT: All important harms or unintended effects in each group**		
To the best of our knowledge, we are not aware of any harms or unintended effects.		
**19-i) Include privacy breaches, technical problems**		
No technical problems or privacy breaches occurred in this study		
**19-ii) Include qualitative feedback from participants or observations from staff/researchers**		
No qualitative feedback was collected from participants during the trial.		
DISCUSSION		
**20) CONSORT: Trial limitations, addressing sources of potential bias, imprecision, multiplicity of analyses**		
**20-i) Typical limitations in ehealth trials**		
Yes. "Although the use of screen media diaries helped to minimize recall bias, self-reporting by mothers could have been influenced by social desirability in underreporting their child's screen time and over-embellishing their parenting capabilities, thus raising issues about the fidelity of the outcomes."		
**21) CONSORT: Generalisability (external validity, applicability) of the trial findings**		
**21-i) Generalizability to other populations**		
Yes. "In view of the proven benefits of screen time reduction, this intervention should be implemented in the primary healthcare setting to educate parents, as well as incorporated into the child health wellness program in preschools and daycare facilities. "Furthermore, future studies should also be expanded to other study populations from middle or higher socioeconomic backgrounds."		
**21-ii) Discuss if there were elements in the RCT that would be different in a routine application setting**		
Yes. "This intervention is recommended to be implemented at the government primary care settings, preschools as well as day-care facilities as part of their child health wellness program. Nevertheless, certain factors such as time and human resources need to be considered. Staff implementing the intervention would also needs to undergo training".		
**22) CONSORT: Interpretation consistent with results, balancing benefits and harms, and considering other relevant evidence**		
**22-i) Restate study questions and summarize the answers suggested by the data, starting with primary outcomes and process outcomes (use)**		
Yes. "This trial investigated the effect of the 'Stop & Play' health education intervention in reducing screen time among preschool children. It is an intervention developed based on the SCT using whiteboard animation videos, infographics reinforcement as well as problem solving session using WhatsApp platform. Overall, the results indicated that it was successful in reducing preschoolers' screen time and improving parental secondary outcomes."		
**22-ii) Highlight unanswered new questions, suggest future research**		
Yes. "future studies should also be expanded to other study populations from middle or higher socioeconomic backgrounds. It is also recommended for future studies to apply mediation analysis to investigate the extent to which the changes in a child's screen time can be attributed to changes within the secondary outcomes. More importantly, the sustainability of this intervention must be ascertained by evaluating the effectiveness of interventions at longer intervals."		
Other information		
**23) CONSORT: Registration number and name of trial registry**		
Yes. "Thai Clinical Trial Registry TCTR20201010002; https://www.thaiclinicaltrials.org/show/TCTR20201010002"		
**24) CONSORT: Where the full trial protocol can be accessed, if available**		
Yes. "Thai Clinical Trial Registry TCTR20201010002; https://www.thaiclinicaltrials.org/show/TCTR20201010002"		
**25) CONSORT: Sources of funding and other support (such as supply of drugs), role of funders**		
Yes. "This study is not funded by any organization"		
**X26-i) Comment on ethics committee approval**		
Yes. "This research has been approved by the Ethics Committee for Research Involving Human Subjects University Putra Malaysia (JKEUPM-2020-284)"		
**x26-ii) Outline informed consent procedures**		
Yes. "Online consent was obtained from both representatives of the cluster (preschools) as well as individual participating mothers before randomization. All participants were free to withdraw from the study at any given time for any given reason and they would not be replaced.		
**X26-iii) Safety and security procedures**		
Yes. "All participants were free to withdraw from the study at any given time for any given reason and they would not be replaced. No personal information was requested through the WhatsApp group and their privacy and confidentiality were protected throughout the study.		
**X27-i) State the relation of the study team towards the system being evaluated**		
Conflict of interest: None declared.		
